# American local government elections database

**DOI:** 10.1038/s41597-023-02792-x

**Published:** 2023-12-19

**Authors:** Justin de Benedictis-Kessner, Diana Da In Lee, Yamil R. Velez, Christopher Warshaw

**Affiliations:** 1 John F. Kennedy School of Government, Harvard University, Cambridge, USA; 2 Department of Political Science, Columbia University, New York, USA; 3 Department of Political Science, George Washington University, Washington, USA

**Keywords:** Politics, Government

## Abstract

The study of urban and local politics in the United States has long been hindered by a lack of centralized sources of election data. We introduce a new database of about 78,000 candidates in 57,000 electoral contests that encompasses races for seven distinct local political offices in most medium and large cities and counties in the U.S. over the last three decades. This is the most comprehensive publicly-available source of information on local elections across the country. We provide partisan and demographic information about candidates in these races as well as electoral outcomes. This new database will facilitate a myriad of new research on representation and elections in local governments.

## Background & Summary

One of the most persistent challenges in the study of urban and local politics in the United States is the lack of information about local elections, candidates, and elected officials^1,2^. As a result, studies on local elections tend to focus on a single time period^3^, geographic unit^4^, or office^5^, rather than holistically examining variation across time, geography, and offices.

In this paper, we describe a new database of election returns from about 78,000 unique candidates in about 57,000 contests in 1,747 cities, counties, and school districts from 1989–2021. Our database is the most comprehensive publicly-available source of information on local elections across the entire country. It includes information about elections for mayors, city councils, county executives, county legislatures, sheriffs, prosecutors, and school boards. It also includes a host of supplemental data, including estimates of candidate partisanship, gender, race/ethnicity, and incumbency status. For many elections, it also includes information on the political characteristics of constituencies, such as their ideology and presidential voting patterns.

This new database will enable scholars to study a wide variety of research questions. It enables examination of whether politicians represent the demographic, partisan, and ideological characteristics of their constituents^6–8^. It also enables expanded work on the factors that affect local elections^9–11^. Moreover, it facilitates study of the incumbency advantage across election types, institutional contexts, and candidate characteristics^12,13^. Finally, this database enables scholars to expand the study of how elections shape a host of political outcomes such as policy^5,14–18^, political communication^19^, interest group activity^20,21^, and intergovernmental lobbying^22^.

## Methods

This section describes how we constructed our database. Our target universe was all cities and counties with more than 50,000 people in the 2020 Census. There are 1,005 counties and 877 cities in our target universe. But many of these cities, especially in California, do not elect mayors, and most counties do not elect executives. Our data collection for school boards was more opportunistic. We also included district attorney (prosecutor) elections that had districts spanning multiple counties which we were not able to match to Census data. The database includes information on the vast majority of the cities and counties in our target universe. First, we describe how we assembled the raw election returns. Next, we describe how we appended supplemental data on candidates race/ethnicity, gender, and partisanship. Lastly, we discuss how we assembled supplemental data on the constituencies of many of the candidates in our database.

### Election returns

The foundation for our data on election returns is previous work on mayoral elections^5,14,15^, county legislative elections^16^, sheriff elections^18,23^, prosecutor elections^24,25^, the MIT Election and Data Science’s Lab’s data on recent elections^26^, and the California statewide election database^27^. We built upon these datasets using several approaches. First, we expanded both the types of offices covered and the temporal coverage of these datasets. We worked with a team of research assistants who coded results from thousands of local elections based on city and county websites. In addition, we scraped data from the crowdsourced website OurCampaigns.com, statewide election websites where available, and some unofficial returns from newspaper archives. Where data sources overlapped in their coverage and conflicted, we prioritized administrative government records as the canonical source, then previously published datasets, then OurCampaigns.com, and then newspapers. These conflicts most likely occurred due to discrepancies between incomplete or provisional and official or final election results that are sometimes released at different times.

**Fig. 1:**
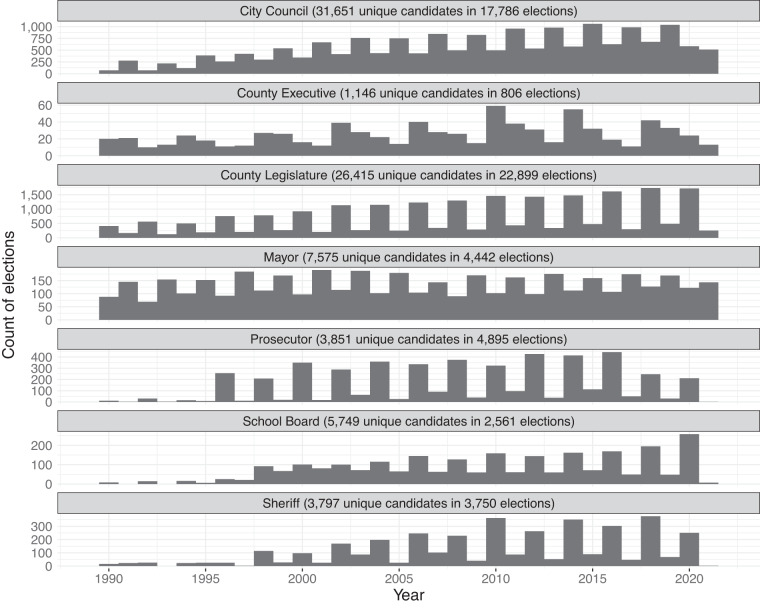
Temporal Coverage of Elections Data.

**Fig. 2:**
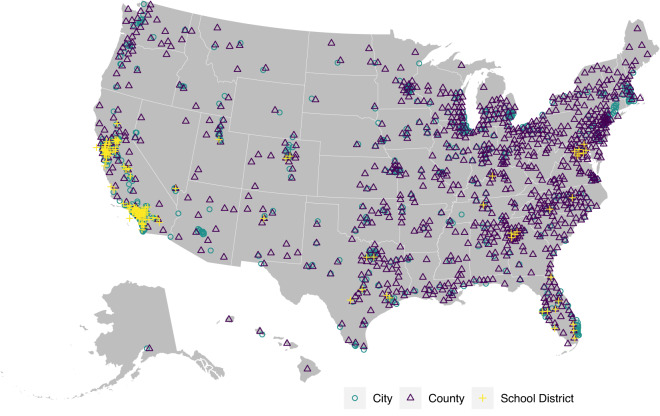
Map of Elections Data.

The resulting dataset of local election returns includes information on 57,139 contests and 77,853 unique candidates in 1,747 cities, counties, prosecutor districts, and school districts from 1989–2021 (Table 1 and Figs. 1, 2). It includes information about elections for mayors, city councils, county executives, county legislatures, sheriffs, prosecutors, and school boards. In many cases, we verified the validity of the election returns by cross-checking them across sources.

**Table 1:** Summary Information about Database.

Office	Years Available	Geographic Units	Elections	% Contested	Unique Candidates
Mayor	1989–2021	578	4,442	80%	7,575
City Council	1989–2021	480	17,786	78%	31,651
County Executive	1989–2021	126	806	78%	1,146
County Legislature	1989–2021	564	22,899	63%	26,415
Sheriff	1989–2021	782	3,750	50%	3,797
Prosecutor	1989–2021	1,598	4,895	26%	3,851
School Board	1990–2021	138	2,561	89%	5,749

### Supplemental information on candidates

We augmented the raw election returns with an array of supplementary information about individual candidates, including their partisanship (even in officially nonpartisan elections), gender, race/ethnicity, and incumbency status. In order to do this, we matched the election returns with a wide range of auxiliary data that enables us to estimate candidates’ partisanship, race/ethnicity, and gender. First, we sought to match each candidate to a record in two national voter files by name and location. Second, we sought to match each candidate with campaign finance-based ideology scores^28^. Third, we matched candidates that served in Congress or state legislatures to determine their party and roll-call based ideal points. We also matched many candidates in recent elections with their Ballotpedia profiles and Reflective Democracy Campaign data (https://wholeads.us/datasets/). Finally, we matched candidates with pictures we obtained from the Internet where possible.

Based on these data, we use Random Forests to predict the race/ethnicity, gender, and party identification of candidates in the data^29^. Random Forest is a collection of identically distributed classification trees, where each tree is comprised of a bootstrap sample from the training set and is grown using a recursive splitting rule that minimizes prediction error. To further reduce the correlation among decision trees, only a fraction of randomly selected covariates are used in each tree during the recursive splitting. Once the set of decision trees has been grown on the bootstrapped samples, the unsampled cases from the test set (“out-of-bag” sample) are used to generate predictions. In particular, the predicted probabilities for each class is generated based on the classification from the collection of decision trees and the final predicted class is generated based on a majority vote-i.e., the most frequent class.

Relative to other statistical learning approaches, Random Forests yield several desirable properties including high accuracy, robustness to outliers and noise, internally unbiased estimate of the generalization error, efficient computation, and the ability to handle many predictors. For example, comparing Random Forest with different versions of logistic regressions in class-imbalanced data, previous work finds that Random Forest provides significantly more accurate predictions in out-of-sample data than any of the logistic models^30^. We also ran several alternative models including ridge and lasso regularizations of generalized linear models, gradient boosting machines (GBM), as well as a super-learner, an ensemble method that accounts for both generalized linear and tree-based models^31^. We confirmed that Random Forest generates much higher accuracy rates than any of the generalized linear models and yields near-identical accuracy rates as GBM and super-learner.

For partisanship, we collapsed our election data by name, geography, and office, which resulted in approximately 61,894 unique observations (only including data with some information on partisanship). Roughly 49% of them (*N* = 30,115) had true party identification categorized as either Democrat or Republican (coded as a 0 or 1 for *prob_democrat*). These are based on ballot returns for either the office in our data or, in a few cases, another office. We use this variable as our target outcome to train a Random Forest for binary classification with 10-fold cross-validation scheme. For model features, we use potentially noisy indicators from the voter file, campaign finance data^32^, the Reflective Democracy Campaign, and partisanship from other sources (e.g., Ballotpedia).

For race/ethnicity, collapsing our data by name, city, and office resulted in 75,591 unique observations (only including data with some information on race/ethnicity). Roughly 18% of them (*N* = 13,671) had true racial identification collected from various external sources, including official lists from non-profit organizations and human-labeled data from Amazon’s Mechanical Turk (coded as a 0 or 1 for *prob_black* and other race/ethnicity categories). Similar to the racial groups used in the Census Bureau and other commonly used prediction methods^33^, we divide race into five categories: White, Black, Latino, Asian, and Other. We use this variable as our target outcome to train a Random Forest for multi-class classification with 10-fold cross-validation scheme. For model features, we use information from the voter files, surname-based Bayesian racial predictions^33^, first and last name-based Bayesian predictions^34^, and predictions from a pre-trained convolutional neural network model based on images of public officials^35^. This approach produces more accurate predictions of race and ethnicity in our sample than existing methods that rely on names and/or geography alone^36^.

For gender, collapsing our data by name, city, and office resulted in 77,352 unique observations (only including data with some information on gender). Roughly 13% of them (*N* = 10,438) had true gender identification collected by the Reflective Democracy Campaign (coded as a 0 or 1 for *prob_female*). We use this variable as our target outcome to train a Random Forest for binary classification with 10-fold cross-validation scheme. For model features, we use information from the voter files, first-name-based gender predictions^37^, and gender estimates in the DIME database^32^.

Overall, our approach generated a probabilistic estimate of most candidates’ partisanship, race/ethnicity, and gender that can be used to study representation, elections, and policymaking.

### Constituency-level data

We augmented the election returns with a variety of information about many candidates’ constituencies. We included information about the ideological preferences of each city and county in our dataset^8^. We also included recent presidential election results for most cities and counties^38–40^. In addition, we assembled a new collection of shapefiles for many city council and county legislative districts. This enabled us to estimate presidential election returns in many local governments’ district-level constituencies by overlaying precinct-level presidential returns on top of the district shapefiles^41^.

## Data Records

The complete American Local Government Elections Database is available for download on OSF^42^. The dataset can be accessed in different formats such as comma-separated files (.csv, for easy access in programs such as Stata, R, Python, or Excel) and in compressed R data files (.rds, for easy access using the R programming language).

The dataset includes two sets of files. First, we include candidate-level data (ledb_candidatelevel.rds and ledb_candidatelevel.csv), in which each observation is a candidate running in a discrete contest along with associated information about that candidate and electoral contest as well as its results. We provide a number of variables at the candidate level (Table 2).

**Table 2:** Candidate-level variables.

Name	Meaning
ledb_candid	Unique candidate identifier.
full_name	Full candidate name. Generally based on the official election returns.
fips	FIPS code for local government.
geo_name	Name of local government.
state_abb	State abbreviation of local government.
office_consolidated	Office candidate ran for.
year	Year of election.
month	Month of election.
district	District candidate ran in. For at-large districts, we create synthetic identifiers that assume a 4-year term.
contest	Unique electoral contest, formed from a combination of **fips, year, month, geo_name, state_abb, office_consolidated**, and **district**.
votes	The number of votes received by each candidate.
vote_share	The candidate’s vote share in the election.
n_winners	The number of winners for each seat. In single-member districts (SMDs), this will be 1.
winner	Whether the candidate won the election.
incumbent	Whether the candidate is an incumbent. We assign incumbency status by matching candidates across contest-years within a given office and place (i.e. city, county, or school district) using a probabilistic name-matching process implemented using the fastLink package in R^58^. This variable is missing in the first 4 years in which we have election data in each individual place since we could not determine whether candidates were new (non-incumbents) vs. incumbents without a previous election cycle.
ballotpedia_url	URL to candidate’s page on Ballotpedia.org (if available).
bonica.cid	Unique candidate identifier in the DIME campaign finance contributor data^28,32^.
contributor.cfscore	Campaign-finance based ideology estimate (CF-Score)^28,32^.
prob_democrat	Probability that a candidate is a Democrat. In partisan elections, candidate partisanship is based on official election returns. In non-partisan elections, we produce a probabilistic estimate of whether each candidate is a Democrat or Republican.
prob_republican	Probability that a candidate is a Republican. In partisan elections, candidate partisanship is based on official election returns. In non-partisan elections, we produce a probabilistic estimate of whether each candidate is a Democrat or Republican.
pid_est	A probabilistic estimate of the best partisan category for each candidate.
prob_female	A probabilistic estimate of whether a candidate is female.
prob_male	A probabilistic estimate of whether a candidate is male.
gender_est	Our estimate of whether someone is male or female.
prob_black	A probabilistic estimate of whether candidate is Black.
prob_white	A probabilistic estimate of whether candidate is White.
prob_hispanic	A probabilistic estimate of whether candidate is Latino.
prob_asian	A probabilistic estimate of whether candidate is Asian-American.
prob_other	A probabilistic estimate of whether candidate is in another race category (e.g., American Indian or Alaska Native).
race_est	A probabilistic estimate of the best race/ethnicity category for each candidate.

Second, we include constituency-level data, in which each observation is at the level of a government jurisdiction. These include data for cities (cities_constituency_data.csv), counties (counties_constituency_data.csv), school districts (schools_districts_constituency_data.csv), city council districts (city_council_districts_constituency_data.csv), and county legislative districts (county_leg_districts_constituency_data.csv). These are available for nearly all cities and counties as a whole. We also have them available at the city council district-level in about 150 cities and the county legislative district level in about 130 counties (Table 3).

**Table 3:** Constituency-level variables.

Name	Meaning
fips	FIPS code for local government.
state	Name of state government.
geo_name	Name of local government.
geo_type	Type of local government.
district	District.
population_2020	Population, based on the 2020 Census.
percent_white	Percent White, based on the 2019 5-year ACS.
percent_black	Percent Black, based on the 2019 5-year ACS.
percent_hispanic	Percent Hispanic, based on the 2019 5-year ACS.
percent_asian_american	Percent Asian-American, based on the 2019 5-year ACS.
mass_ideology_2020	A cross-sectional measure of the mass public’s ideology in 2020^59,60^. Only available at the city and county-level.
pres_pctD_08	Presidential vote shares based on precinct-level data on the 2008 presidential vote^38^.
pres_pctD_16	Presidential vote shares based on precinct-level data on the 2016 presidential vote^26,39^.
pres_pctD_20	Presidential vote shares based on precinct-level data on the 2020 presidential vote^26,40^.

## Technical Validation

In this section, we discuss a number of technical validations of our data. We validate a number of aspects of the candidate-level data, including the vote totals for individual candidates and our imputations for candidates’ partisanship, race/ethnicity, and gender.

### Validation of election data

We validate our elections data by comparing the consistency of candidate’s vote totals across secondary sources, and obtaining administrative data where possible.

### Validation of estimates of partisanship, race, and gender

A key contribution of our database is a set of estimates for candidates’ partisanship, race/ethnicity, and gender. In some cases, these are based on observed data on these variables. But in other cases, we use Random Forest algorithms for these variables based on potentially noisy indicators as described above. Thus, it is important to validate our estimates of these variables.

We first validate our Random Forest model of *race/ethnicity* using 20% of the data with outcomes as a test set. Table 4 shows the sensitivity (true positive rate), specificity (true negative rate), precision (positive predictive value), and F-1 score. Note that F-1 score is the harmonic mean of precision and sensitivity-i.e., $2\cdot \frac{precision\cdot sensitivity}{precision+sensitivity}$.

**Table 4:** Validation of Random Forest Classification for Race/Ethnicity.

	Sensitivity	Specificity	Precision	F-1 Score
Overall (100%)	0.930	0.876	0.929	0.929
Asian (3%)	0.833	0.997	0.887	0.859
Black (15%)	0.802	0.982	0.889	0.843
Caucasian (72%)	0.969	0.833	0.939	0.954
Hispanic (10%)	0.866	0.993	0.928	0.896

Note: Total sample size is 2,745.

The F-1 score for our model of race/ethnicity is 0.929, which is significantly higher than other racial classification methods commonly used in academic research^33–35^. Our results also show substantially high sensitivity scores across all racial categories, particularly among Asians and Blacks that are generally associated with high false negative rates^33^. We also examine the Receiver Operating Characteristic (ROC) curves and find the area under the ROC curve (AUC) to be at least as high as 0.97 across all racial categories, indicating an outstanding classification success.

Next, we perform the same validation check for our Random Forest model of *gender* using 20% of the data with outcome as a test set. Table 5 shows the results of our model performance. The F-1 score for our model of gender is approximately 0.993 and the AUC score is above 0.99, indicating a very high predictive ability. We also check whether our gender predictions for the subset of our candidate data that are female mayors (i.e. winning mayoral candidates) against recent estimates of mayoral gender from the Center for American Women and Politics (CAWP)^43,44^. We find that out of 103 candidates in our data that matched to CAWP’s list of women mayors, our random forests models estimate that two are men. This is similar to the accuracy rate reported in Table 5. Through a manual check of CAWP’s lists of 661 unique mayors in 2021 and 2022 who are designated as women, we also find that seven of these mayors were actually men, yielding an accuracy rate of 0.989 - similar to our overall accuracy rate.

**Table 5:** Validation of Random Forests Classification for Gender.

	Sensitivity	Specificity	Precision	F-1 Score
Overall	0.993	0.993	0.993	0.993
Men (75%)	0.993	0.993	0.998	0.995
Women (25%)	0.993	0.993	0.981	0.987

Note: Total sample size is 2,221.

Lastly, we perform the same validation check for our Random Forest model of *partisanship* using 20% of the data with outcome as a test set. Table 6 shows the results of our model performance. The F-1 score for our model of partisanship is approximately 0.903 and the AUC score is above 0.95, indicating a very high predictive ability.

**Table 6:** Validation of Random Forests Classification for Partisanship.

	Sensitivity	Specificity	Precision	F-1 Score
Overall	0.903	0.902	0.903	0.903
Democrat (53%)	0.912	0.893	0.904	0.908
Republican (47%)	0.893	0.912	0.901	0.897

Note: Total sample size is 6,001.

The data files we make available include the final estimated probabilities that each candidate falls in each category of partisanship, gender, and race/ethnicity. This enables scholars to make their own decisions about how to use the results of our imputation models in downstream analyses.

## Usage Notes

As the most comprehensive record of local election outcomes and information about local political candidates, our database presents myriad opportunities for researchers looking to expand knowledge about democracy in subnational politics. There are several general points scholars should consider as they use the data.

First, the data is much more comprehensive than previous datasets on local elections. But the target universe is not totally comprehensive. It only includes cities, counties, and school districts with a population of at least 50,000 in 2020. Moreover, it is missing some elections in this target universe where we were unable to find data. In some cases, there may be raw election data for smaller geographic units available from the sources we discussed earlier^5,18,23–27^.

Second, researchers should note that many prosecutor districts span counties. In addition, one of our raw sources of prosecutor election data included unique numeric district identifiers, but no district numbers or names^25^. This makes it more difficult to determine incumbency status and other characteristics of prosecutor candidates. In cases where we were unable to properly match a contest to its county or district, we include the original district code from the source data and a synthetic fips code constructed by concatenating state codes with the district code from the source data.

Third, as described above, we imputed the partisanship and race of many candidates. This enables scholars to study a wide variety of research questions related to elections and representation. In the data, we provide the predicted probability of these imputations. For applications that study the causes and consequences of individual elections, scholars may choose to only include candidates with high predicted probabilities of being in particular racial, partisan, or gender categories^45^. For example, recent research has examined the close link between partisan voting patterns in national and subnational elections to argue that local races are increasingly nationalized^46,47^. Our database allows scholars to test both the causes and consequences of this nationalization across many different types of elected office. For this analysis, researchers may decide to classify candidates into the partisan group with the highest probability (e.g., only including candidates with known partisanship or ones with >90% in our imputation model).

Scholars could also use our database to examine the aggregate characteristics of candidates and election officials. For instance, an important question in the study of American elections is the under-representation of women and non-white racial groups at various levels of government^7,48–55^. While prior work suggests that women’s under-representation in local governments mirrors their underrepresentation in Congress and state legislatures, and that the representation of women in local government appears to have plateaued over the past two decades, the limits of previous data have prevented researchers from examining the barriers to both gender and racial parity in local politics^6^. Our local elections data enable researchers to assess questions about barriers to both women and racial minorities in politics at the subnational level by vastly expanding the temporal and geographic scope of previous data on local candidates and officeholders, as well as expanding the scope of previous datasets. When estimating the racial, gender, or partisan composition of a particular geographic unit, recent research finds that it is more accurate to average up the predicted probabilities for all individuals within a geographic unit rather than aggregating after assigning each individual to a single category based on the highest predicted probability^56^. Because our data provides the full distribution of predicted probabilities across each category for partisanship, race/ethnicity, and gender, researchers are able to examine topics like representation and turnout while avoiding substantial error in estimating the demographic composition.

**Fig. 3:**
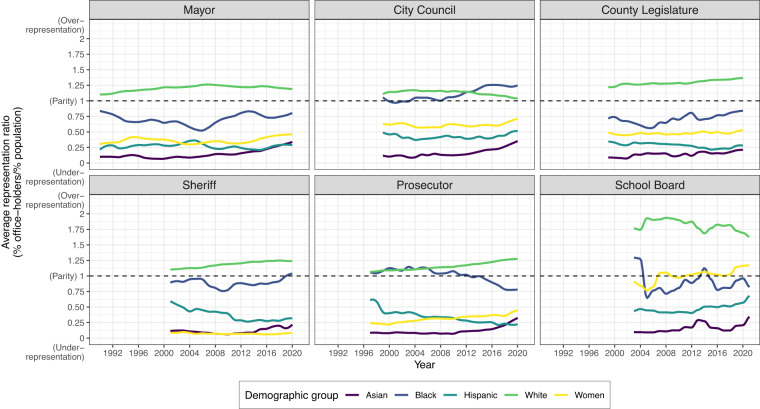
Descriptive representation by office. Lines indicate smoothed local averages of the ratio between the percent of officeholders and the percent of the population in each gender, racial/ethnic group, and are plotted for years after which our data cover at least 20% of the total jurisdictions for which we have some composition data for that office.

A brief examination of over-time patterns of candidates’ race and gender in our data illustrates how it could be used to assess the institutional and contextual determinants of descriptive representation. Figure 3 shows the relative representation of women, Blacks, Hispanics, and Asian-Americans based on the ratio between the share of local officeholders of each type and their fraction of the population. This brief demonstration indicates that women remain under-represented in the majority of local offices, with the percentage of winning candidates under their percentage in the population for all offices except school boards. The breadth of our data allows us to expand this usage beyond single offices or small time spans, and shows that there are dramatic differences in patterns across offices in women’s representation. Women are most under-represented in sheriff elections, and tend to be best represented in school board elections, in line with recent work on California^57^. Similarly, our data on multiple racial groups allows usage of our data to examine under-representation of multiple groups across offices. Figure 3 suggests that Hispanics and Asian-Americans are particularly under-represented and that descriptive representation is especially poor among sheriffs, but that city councils, in particular, consist of Black legislators at rates roughly proportional to population demographics. White officeholders, meanwhile, are overrepresented in every local office across the last three decades.

Researchers may conduct many other analyses using variables described earlier in the manuscript, as well as by combining our data with additional institutional or contextual data. We encourage researchers to fully harness our data to both further describe the heterogeneity in these over-time and between-office trends, as well as further examine the causes and consequences of local elections.

## Data Availability

The replication code for the two demonstrations of our data is publicly available on OSF^42^, and can be used under a CC-BY license.
